# Patient preferences for treatments in hormone receptor-positive/HER2-negative metastatic breast cancer in Italy: a discrete choice experiment study

**DOI:** 10.1186/s12885-025-14308-4

**Published:** 2025-05-22

**Authors:** Grazia Arpino, Carmine De Angelis, Lorenzo Gerratana, Matteo Lambertini, Sarah Igidbashian, Rosanna Gramigna, Xavier Guillaume

**Affiliations:** 1 Department of Clinical Medicine and Surgery, University of Naples Federico II, Naples, Italy; 2 Clinical and Translational Oncology, Scuola Superiore Meridionale, Naples, Italy; 3 Department of Medical Oncology, CRO Aviano, National Cancer Institute, IRCCS, Aviano, Italy; 4 Department of Medicine, University of Udine, Udine, Italy; 5 Department of Medical Oncology, Clinica di Oncologia Medica, IRCCS Ospedale Policlinico San Martino, Genoa, Italy; 6 Department of Internal Medicine and Medical Specialties (DiMI), School of Medicine, University of Genoa , Genoa, Italy; 7 AstraZeneca, Milan, Italy; 8 Daiichi-Sankyo, Milan, Italy; 9 Oracle Life Sciences, Paris, France

**Keywords:** Adverse events, Metastatic HR positive/HER2 negative breast cancer, Stakeholder preferences, Treatment administration, Treatment choice

## Abstract

Hormone receptor (HR) positive (HR +) and human epidermal growth factor receptor 2 (HER2) negative (aka HER2 −) breast cancer (BC) is the most frequently diagnosed subtype. Recent development of next-generation endocrine therapies (e.g. selective estrogen receptor degraders (SERDs); third-generation aromatase inhibitors (AI) and targeted therapies (e.g., CDK4/6, PI3K, and mTOR inhibitors)) as well as antibody drugs conjugates (ADC, eg. T-DXd and SG) showed promising results with meaningful improvements in survival for patients with metastatic HR + HER2 − BC. Therapy selection is mainly based on clinical, tumor pathological and molecular characteristics as well as on efficacy based on trial data, nevertheless, side effect profiles are key differentiators of treatments in the metastatic setting. Therefore, understanding how patients evaluate various treatment attributes and how these change in different clinical situations is fundamental toward the choice of optimal therapeutic strategies for treating metastatic HR + HER2 − Stage IV patients. Here, we investigated treatment preferences of a total of 102 stage IV HR + HER2 − breast cancer patients in Italy by developing and applying a survey instrument based on discrete choice experiment (DCE). Treatment efficacy was the top valued attribute across all patient segments and the second most important attribute was the risk of grade ≥ 3 adverse events (AE). Overall, therapies with better outcomes of PFS or AE grade 3 or higher would have a higher impact on the preference to choose a treatment from a patient perspective.

**Supplementary Information:**

The online version contains supplementary material available at 10.1186/s12885-025-14308-4.

## Background

Breast cancer (BC) is the most frequent cancer among women worldwide, with more than 2.3 million new cases in 2022 [1]. The most recurrent subtype of breast cancer diagnosed is the hormone receptor (HR) positive (HR +) and human epidermal growth factor receptor 2 (HER2) negative (HER2 −) BC, which accounts for two-thirds of all BC diagnoses [2]. Inhibiting HR by means of endocrine therapies (ET) represents the mainstay of treatment for HR + HER2 − BC subtype, both in its early- and advanced stages of development. Approximately 5% of all patients are diagnosed with metastatic stage IV de novo BC, and ~ 20–30% of diagnosed early-stage BC then progresses to metastatic disease, which is a largely uncurable [3]. However, recent development of more effective systemic treatments including next-generation endocrine therapies (e.g. selective estrogen receptor degraders, SERDs; third-generation aromatase inhibitors (AI) and targeted therapies such as CDK4/6, PI3K, and mTOR inhibitors) showed promising results with meaningful improvements in survival [2]. According to the European Society for Medical Oncology (ESMO) guidelines, CDK4/6 inhibitors in combination with endocrine therapy are recommended as the standard of care for the treatment of hormone receptor-positive, HER2-negative advanced breast cancer. This combination has been shown to significantly improve progression-free survival and overall survival compared to endocrine therapy alone [4]. In keeping with this, treatment for HR + HER2 − metastatic disease is essentially focused on initial therapy with CDK4/6 inhibitors (palbociclib, abemaciclib, ribociclib) combinations with oral AIs (anastrozole or letrozole) or SERD (fulvestrant) [2]. The decision depends on a number factors, including: i) patient characteristics (comorbidities, performance status, compliance concerns); ii) biomarker status at least in 2L + treatment (*PIK3CA*mut, *BRCA1/2*mut, HER2-low); iii) sites of metastases (brain, bone, viscera) including number of lesions and symptomatology, iv) patient desire for tolerable therapy, or preference for mode of administration (oral, IV, intramuscular); and v) prior treatment history (prior adjuvant, prior lines) and time to clinical progression across lines of therapy. While efficacy based on trial data plays a large role in therapy selection, side effect profiles are key differentiators of treatments in the metastatic setting and thus it is important to understand how these and other characteristics are considered. Yet, little is known about the specific stakeholder preferences that may underlie real-world treatment decision-making. Understanding how patients evaluate various treatment attributes and how these change in different clinical situations is fundamental toward the choice of optimal therapeutic strategies for treating metastatic ER + HER2 − Stage IV patients.

Here, we investigated treatment preferences of a total of 102 patients with HR + HER2 − metastatic breast cancer in Italy by developing and applying a survey instrument based on discrete choice experiment (DCE).

## Methods

### Study cohort

The study included patients (aged ≥ 18 years) in Italy with self-reported diagnosis of HR + HER2 − metastatic BC currently receiving their first or second line of endocrine therapy, or expecting to receive or currently receiving their first-line chemotherapy, or having completed at least one line of chemotherapy for the treatment of their metastatic disease. Exclusion criteria were: i) unable or unwilling to comply with the requirements for study participation; ii) unable to have access to a mobile device with internet (e.g., smartphone, tablet) or a computer with internet access.

### Study design

Participants were recruited through databases, identification and referral from physician sites, invitations by patient organizations, and social media. The survey instrument and DCE design (i.e., attributes and levels) were specifically created for the survey. The attributes and levels were determined based on literature review and qualitative interviews with patients. The survey was tested with patients in cognitive interviews before data collection started. The study was conducted in two distinct phases, i.e.: i) Phase 1, which was “qualitative” and included 15 HR + HER2 − Stage IV patients, 9 chemotherapy-treated and 6 without chemotherapy exposure; ii) Phase 2, which enrolled HR + HER2 − Stage IV 102 patients aimed to perform a preference survey.

Phase I study was based on a 45-min interview (from April to May 2022) via telephone with computer screen share to understand key treatment attributes and factors influencing treatment decision-making. Participants transcripts were coded using NVivo (v12 Plus; Alfasoft) qualitative data analysis software to identify key themes emerging from the data, including attributes and associated attribute levels to be captured in the preference surveys. Themes were developed inductively through iterative comparison of participant responses until information saturation was achieved. Results from Phase I study were used to inform the DCE administered to patients enrolled in Phase 2 study. In particular, Phase I allowed to select the following attributes for further quantification of their preference weights in a DCE: progression-free survival (PFS), risk of neutropenia, risk of alopecia, risk of vomiting, risk of diarrhea, risk of grade 3/4 adverse events (AEs) and mode of administration. Four additional interviews (from June to July 2022) were performed to confirm our findings and recommendations of attributes, allowing us to move on to i) the cognitive interview step aimed to ensure that key attributes were included, and item wording was clear, appropriate, and understood as intended, and ii) the subsequent quantitative study (Phase 2).

Phase 2 of the study was based on a 25-min online survey (from March to July 2023) using a 2 DCEs modeling approach (i.e., one for first-line treatments and one for second-/third-line treatments) designed to understand how patients may value different applicable treatments given their advantages and disadvantages relative to each other, and also which factors may influence how patients may value different treatments (such as the number of therapy lines received for their metastatic breast cancer and side-effects they experienced because of treatments). To help familiarize respondents with the DCE attributes and levels prior to completing the choice tasks, they were asked to rate the levels for each attribute on a scale from 1 = very bad to 5 = very good. The Root Likelihood (RLH) the method was used to assess the consistency of each participant’s answers to the DCE. Briefly, RLH is the geometric mean of the respondents’ likelihood (i.e., the *nth* root of the likelihood where *n* is the number of choice tasks per respondent) which represents the fit of the choice model on the respondents’ choices. A null model with equal probabilities for all choice options (i.e., random choices) has an RLH of *1/k*, with *k* meaning the number of choice options per choice tasks [5].

### Statistical analysis

Sociodemographic characteristics (i.e., age, gender, working status, education, living area), cancer history and experience (i.e., time since breast cancer, current stage, treatments, AEs related to treatments, hospitalization due to AEs, treatment stopped because of AEs) were collected. Descriptive statistics (means, standard deviations [SDs], medians, and interquartile ranges [IQR] for continuous variables and percentages for categorical variables) were calculated for all questions in the survey for the total sample. This analysis of the distributions was a part of testing of model assumptions and helped inform the appropriate modeling techniques. For the DCE analysis, a hierarchical Bayesian logistic regression model was used to analyze the data obtained [6]. This led to the calculation of relative preference weights for each attribute level at an individual level, from which relative importance of each attribute were derived. Mean utility weights were calculated in an aggregated manner which provided the relative preference for a specific attribute level in order to understand the difference across levels within each attribute [6]. To know the contribution each attribute made to the total utility of a treatment profile, the relative importance was calculated, by dividing the range of each attribute (utility of the most favorable level minus utility of the least favorable level) by the sum of the ranges of all attributes and multiplying by 100 to convert to a percentage. The resulting estimates reflected the importance of each attribute, relative to the others. Importance estimates were ratio-scaled; an attribute with an importance of 20% was twice as important as an attribute with an importance of 10%.

## Results

### Patient characteristics

A total of 130 patients were enrolled for the Phase 2 survey. When we applied the Root Likelihood (RLH) method to assess the consistency of each participant’s answers to the DCE, 28 patients were identified with inconsistent answers and were not included in the final sample. In particular, 21 patients were removed from study due to suspected duplication; 2 patients were excluded because of inconsistent answers to non-DCE questions; 5 patients have been excluded because their answers to the DCEs were inconsistent as per the RLH method.

Patient characteristics (*N* = 102) are shown in Table 1. The median age was 56.0 (IQR: 47.0–63.0) years. Most were married/living with a partner (75%), had completed at least high-school/college/postgraduate education (81.0%), were employed (51.0%), lived in an urban area (74%) with the remaining living in suburban (~ 19%), or rural areas (~ 7%). One third of the population had at least one minor people in their household. Among them, more than 90% had at least one child in their household. Most of the patients (75%) were diagnosed with BC within the past 4 years and with Stage IV BC within the past 2 years. More than 80% of the study population had already received endocrine therapy. At the time of the study, most of the patients (*N* = 99; 97%) were treated for BC. Hormone therapy was the current treatment most frequently cited by study participants (59%), followed by chemotherapy (39%) and targeted therapy (14%). Only three patients were untreated, but all were awaiting chemotherapy (100%) (Table 1). Among patients treated by chemotherapy (75%), the majority received 1 course of chemotherapy (53%), with paclitaxel and capecitabine the two most frequent drugs (24% and 25%, respectively) to treat patients when BC became metastatic (Table 1). Regarding patients treated by hormone therapy (HT) (76%), anastrozole and letrozole were the two most prevalent HT drugs (34% and 33%, respectively) given to patients when BC became metastatic (Table 1). When surveying AEs experienced by BC patients, vomiting was the most common AE due to treatment (65%), followed by alopecia (63%), neutropenia (54%) and diarrhea (49%). Only a minority of patients reported to have been hospitalized (11%) and stopped a treatment (9%) due to AEs. Most participants (54%) were restricted in physically strenuous activity, but could walk, were able to carry out light housework and were menopausal because of age.

**Table 1:** Patient characteristics

	***N* = 102**	
**Age [yrs]**		
median (Q1;Q3)	56 (47;63)	
min–max	47–83	
**Current marital status**		
Married/living with partner	74.5%	76
Single	22.5%	23
Decline to answer	2.9%	3
**Education level**		
Less than high-school	14.7%	15
High-school	41.2%	42
College/university	26.5%	27
Postgraduate	13.7%	14
Decline to answer	3.9%	4
**Employment status**	*Before BC diagnosis*	*Currently*
Employed Full time	41.2% 42	22.5% 23
Employed Part time	17.6% 18	15.7% 16
Leave of absence	2.0% 2	12.7% 13
Unemployed	18.6% 19	18.6% 19
Retired	12.7% 13	22.5% 23
Other	7.8% 8	7.8% 8
**Living area**		
Urban	74.5%	76
Suburban	18.6%	19
Rural	6.9%	7
**Number of minor people in the household**		
No minor people	70.6%	72
1 minor person	16.7%	17
2 minor people	8.8%	9
3 minor people	3.9%	4

Percentages could not add up to 100% due to rounding

### Patients preferences

**Fig. 1:**
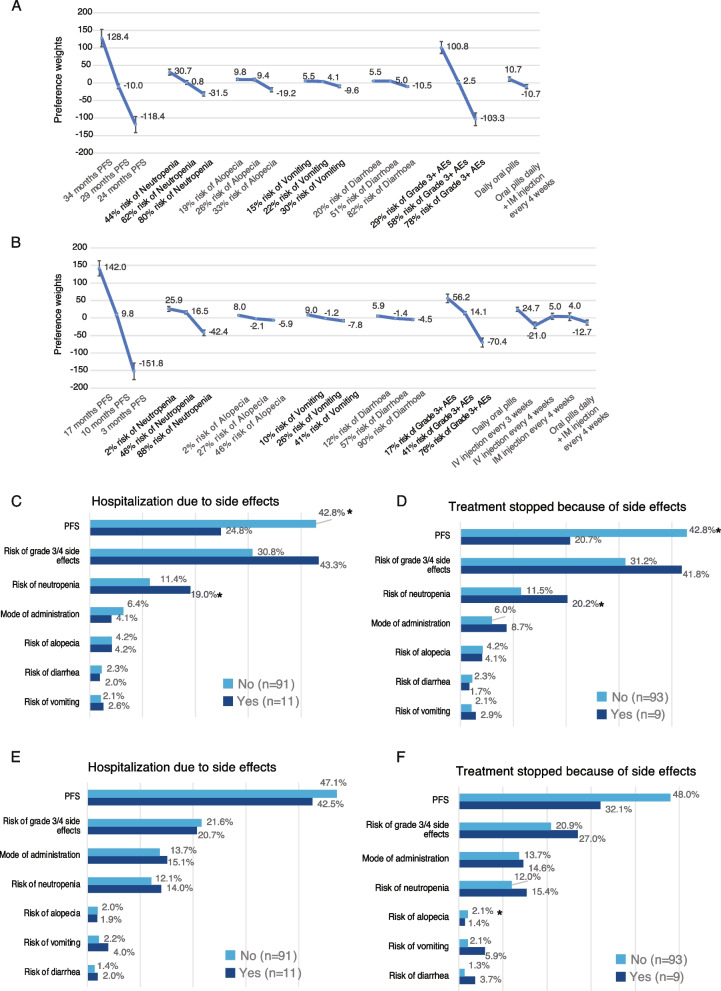
**A** Attribute-level preference weights for first line scenario (1L). Bars represent the 95% CI for each attribute. Abbreviations: (PFS) progression free survival; (AE) adverse event; (IM) intramuscular injection. **B** Attribute-level preference weights for second line (or more) scenario (2L +). Bars represent the 95% CI for each attribute. Abbreviations: (PFS) progression free survival; (AE) adverse event; (IM) intramuscular injection. **C** Bar plot indicating the Relative Importance (RI) of treatment attributes in the case of “Hospitalization due to AEs” for 1L scenario. Light blue bars, patients who have never been hospitalized; dark blue bars, patients who had already been hospitalized. Numbers shown are the relative importance percentage for each attribute. Asterisks indicate significant difference between groups (*p* < 0.05; Student’s t-test). N, number of patients. **D** Bar plot indicating the Relative Importance (RI) of treatment attributes in the case of “Treatment stopped because of AEs” for 1L scenario. Light blue bars, patients who have never been hospitalized; dark blue bars, patients who had already been hospitalized. Numbers shown are the relative importance percentage for each attribute. Asterisks indicate significant difference between groups (*p* < 0.05; Student’s t-test). N, number of patients. **E** Bar plot indicating the Relative Importance (RI) of treatment attributes in the case of “Hospitalization due to AEs” for 2L + scenario. Light blue bars, patients who have never been hospitalized; dark blue bars, patients who had already been hospitalized. Numbers shown are the relative importance percentage for each attribute. Asterisks indicate significant difference between groups (*p* < 0.05; Student’s t-test). N, number of patients. **F** Bar plot indicating the Relative Importance (RI) of treatment attributes in the case of “Treatment stopped because of AEs” for 2L + scenario. Light blue bars, patients who have never been hospitalized; dark blue bars, patients who had already been hospitalized. Numbers shown are the relative importance percentage for each attribute. Asterisks indicate significant difference between groups (*p* < 0.05; Student’s t-test). N, number of patients

Attribute preference weights level by patients for 1L) are shown in Fig. 1A. The difference between level estimates allowed for the evaluation of the effect of a change within an attribute. Increasing PFS from 24 to 36 months reached the higher preference weights than lowering the risk of all considered AEs (i.e., neutropenia, alopecia, vomiting, diarrhea). Increasing PFS followed closely by lowering the risk of AEs grade 3 or higher from 78 to 29% were the most important attributes to 1L (Fig. 1A; Figure S1A). Risk of neutropenia was the third most important attribute followed by the mode of administration (daily oral pills vs. oral pills daily + IM injections) and the risk of each other specific AE, with the risk of vomiting was the least important. Therapies offering better PFS outcomes or therapies that lower the risk of developing AEs grade ≥ 3 would have a higher impact on the preference to choose a treatment from a patient perspective (Fig. 1A; Figure S1A). Patients who had never been hospitalized due to AEs attributed more importance to PFS than those who had been hospitalized (42.8% vs. 24.8%, *p* < 0.05, Student’s t-test; Fig. 1C). Those patients attributed less importance to the risk of neutropenia than those who had been hospitalized (*p* < 0.05; Student’s t-test; Fig. 1C). On the other hand, patients who had already been hospitalized tend to attribute greater importance to the risk of grade ≥ 3 AEs (43.3% vs. 30.8%; *p* = 0.07; Student’s t-test; Fig. 1C). This may be the result of their hospitalization due to AEs. Patients who have never stopped treatment because of AEs also attributed more importance to PFS than those who have interrupted treatment (42.8% vs. 20.7%; *p* < 0.05; Fig. 1D). In contrary, patients who have interrupted treatment gave more importance to the risk of neutropenia (20.2% vs. 11.5%; *p* < 0.05 Student’s t-test) and tend to attribute greater importance to risk of grade ≥ 3 AEs (41.8% vs. 31.2%) (Fig. 1D). This may be interpreted as the negative experience of discontinuing treatment impacting patient preferences.

Attribute preference weights level by patients for 2L (or more) (2L +) are shown in Fig. 1B. PFS was the most important treatment feature followed by the risk of AEs grade ≥ 3. The importance of PFS was higher vs. 1L DCE, as well as the mode of administration, which was now ranked as the third most important feature, driven by the multiple options presented to respondents (Fig. 1B; Figure S1B). No significant difference in preferences for 2L + treatments was observed between patients who had previously been hospitalized due to AEs and those who had not (Fig. 1E). Patients who had experienced AEs leading to hospitalization gave much more importance to PFS in 2L + versus 1L (42.5% vs. 24.8%; Fig. 1C and E, respectively), indicating that they may be more willing to value most effective treatments in 2L + than in 1L, even if they have significant AEs. These patients may understand that getting 2L + treatments means a more serious disease calling for the most effective treatments available. Although not significant, patients who never have stopped treatment due to AEs gave more importance to the PFS (Fig. 1F).

Lastly, we combined preference weights of respondents with clinical outcomes derived from the literature for therapies administered in 1L and 2L + settings (Table S1-2), to calculate preference scores for estimating the patient preference for the different treatments available (Tables 2 and 3). In the 1L setting, ribociclib/fulvestrant was defined as the most often preferred treatment by patients versus other treatments available (Tables 2 and 3). This may be explained by the differences in efficacy and risk of grade ≥ 3 AEs. In the 2L + setting, ribociclib/fulvestrant was observed as the most preferred treatment, followed by abemaciclib/fulvestrant as the second most preferred option (Table 4).

**Table 2:** Preference scores for estimating the patient preference for the different 1L treatments available

1L DCE (*n* = 102)	Mean	SD	Min	Max
Ribociclib/fulvestrant	215.7	109.7	-150.7	355.1
Abemaciclib/NSAI	-6.7	70.7	-196.9	199
Ribociclib/letrozole (CompLEEment-1)	-138.2	103.8	-280.7	207.3
Palbociclib/letrozole	-153.8	92.8	-303.9	188.3
Ribociclib/goserelin/ (tamoxifen or NSAI)	-209.5	121.4	-350	183.8
Ribociclib/letrozole (MONALEESA-2)	-232	82.9	-330.6	61.4

*NSAI* Non-Steroidal Aromatase Inhibitor

**Table 3:** Preference scores for estimating the patient preference for the different 2L + treatments available

2L + DCE (*n* = 102)	Mean	SD	Min	Max
Ribociclib/fulvestrant	112.1	106.5	-194.6	218.9
Abemaciclib/fulvestrant	96.5	145.8	-223.8	273.8
Fulvestrant post-menopausal (MONALEESA-3)	91.4	111.5	-243.4	292.6
Fulvestrant post-menopausal (MONARCH-2)	75.7	87.3	-235.9	228.7
Everolimus/exemestane	33.8	55.4	-123.7	206.4
Olaparib	13.4	67.6	-140.6	217.9
Talazoparib	5.9	47.1	-146.9	174.4
Trastuzumab deruxtecan	-21.2	60.1	-157.9	250.8
Fulvestrant pre-menopausal	-27.8	164.5	-301.6	323.7
Palbociclib/fulvestrant	-78.08	84.1	-256.4	175.8
Sacituzumab govitecan	-190.2	121.6	-336.1	326.5

**Table 4:** Treatment characteristics over the cohort of patients enrolled (*N* = 102)

	***N* = 102**
**Course of chemotherapy received**	
1	52.9% 54
2	13.7% 14
3 or > 3	7.8% 8
Never	25.5% 26
**Type of chemotherapy drugs since metastatic BC**	*(N* = *76)*
Capecitabine	23.7% 18
Eribulin	5.3% 4
Gemcitabine	14.5% 11
Paclitaxel	25.0% 19
Vinorelbine	3.9% 3
Other	28.9% 22
Do not know	14.5% 11
**Course of HT received**	*(N* = *76)*
1	56.6% 47
2	22.9% 19
3 or > 3	13.3% 11
Never	7.2% 6
**Type of HT drugs**	*(N* = *77)*
Anastrozole	33.8% 26
Exemestane	14.3% 11
Letrozole	32.5% 25
Fulvestrant	20.8% 16
Other	2.6% 2
Don't know	6.5% 5
**AEs ever experienced**	
Neutropenia	53.9% 55
Alopecia	62.7% 64
Vomiting	64.7% 66
Diarrhea None of these	49.0% 50 6.9% 7

Percentages could not add up to 100% due to rounding

*HT* hormonal therapy

## Discussion

The attractiveness of a treatment to individuals depends on their relative preferences for these attributes expressed by their willingness to accept trade-offs among them. Although there are alternatives to eliciting patient preferences (e.g., revealed preferences, direct ratings/rankings, etc.), DCE is one of the most common approaches for assessing preferences in a healthcare context and has been increasingly applied in health care decisions. DCE has a strong foundation in psychology and economics [7]. This approach is based on the principle that treatment options can be assigned to attributes of interest. The attractiveness of a treatment to individuals will depend on their relative preferences for these attributes, as expressed by the frequency with which they choose profiles with the preferred features; these values are then used to estimate the predictive probabilities that determine whether respondents will be more likely to choose one hypothetical treatment over another. Although there are alternatives for eliciting preferences (e.g., revealed preferences, direct ratings/rankings, etc.), DCE is one of the most common approaches for assessing preferences in a healthcare context, and it has become of increasing interest to health authorities and clinicians to facilitate shared decision-making [8]. Each hypothetical treatment profile consisted of combinations of characteristics (“attributes”) and “levels” reflecting the attributes for each hypothetical treatment profile. Respondents’ selections allowed for an assessment of the trade-offs they were willing to make between positive and negative aspects of the treatment profiles.

We used a balanced, overlap design to select which combination of levels were shown to a respondent. The DCE method is superior to other approaches because it mimics a real-world situation where a medication’s attributes do not occur in isolation. For example, one must weigh both efficacy and toxicities simultaneously among available therapies when selecting treatment. Here, we adopted a two DCEs modeling approach (i.e., for 1L and 2L/3L treatments) designed to understand how patients may value different applicable treatments given their advantages and disadvantages relative to each other and which factors influence how patients may assess different therapeutic strategies.

We found that efficacy was the top valued attribute across all patient segments. The relative importance of efficacy was about ~ 10% greater in 2L + setting vs. 1L setting. The second most important attribute is the risk of grade 3–4 AEs which demonstrate how patients were fully aware of the utility they place on avoidance of grade 3–4 AEs. Interestingly, the importance of grade 3–4 AEs attribute was greater in 1L than in 2L + . This may be explained by the increasing importance of effective treatment as the disease progresses. Overall, therapies with better outcomes of PFS or AE grade 3 or higher would have a higher impact on the preference to choose a treatment from a patient perspective than those that have a worse PFS and AE grade 3 or higher but with lower risk of AEs or preferred dosing regimens, like pills taken daily. Interestingly, we found that for 1L treatments, patients who have had negative experiences of AEs attach significantly more importance to the risk of grade 3–4 AEs as well as to risk of neutropenia. Prior studies suggested that patients prioritize treatment aspects that can impact their QoL [9] particularly in the metastatic setting, as patients could potentially be less willing to endure uncomfortable treatment regimens with a high risk of toxicity that could negatively impact the quality of time remaining.

Lastly, we found that ribociclib/fulvestrant was most frequently the preferred treatment in 1L setting according to the preference weights of respondents and the clinical outcomes collected from the literature. In 2L + setting, ribociclib/fulvestrant was also the preferred option followed by abemaciclib/fulvestrant. Indeed, in the first-line (1L) setting, ribociclib plus fulvestrant showed the highest progression-free survival (PFS) rate (34%; Table S1) while also reporting the lowest incidence of grade ≥ 3 adverse events (AEs) (29%; Table S1). Similarly, in the second-line or later (2L +) setting, the PFS was the second highest (15%; Table S2), with grade ≥ 3 AEs remaining low at 29%, ranking among the four lowest (Table S2). Notably, recent updates from the phase III MONALEESA-3 trial [10, 11] confirmed that ribociclib plus fulvestrant showed a significant overall survival benefit over placebo plus fulvestrant in patients with ER + HER2 − metastatic BC [11, 12].

In our study, we did not report any potential conflict between patient preferences and clinical practice. However, in the case of conflicts we suggest to implement a strategy to guide shared decision-making (SDM; [13]) based on: i) build trust, ii) clarify the conflict, and iii) explain options clearly. Eventual tools like decision aids and motivational interviewing could be implemented as well as organizing multidisciplinary teams if needed.

Possible limitations of the study are: 1) certain subgroups of patients may be under-represented because either we did not collect any geographic information due to the nature of this study which was based on convenience sampling methods and online recruitment; 2) the reliance on online recruitment which may have excluded subgroups of patients under-represented, such as older patients, less fit patients or those with a lower socio-economic status, institutionalized patients, and those with the most severe comorbidities and disabilities, potentially skewing the sample toward younger, urban populations; 3) a selection bias since our study was an online patient-reported survey, which most likely under-represented people without access to or comfort with online administration such as less healthy elderly people, institutionalized patients, and those with the most severe comorbidities and disabilities; 4) a recall or knowledge bias due to the fact that answers have been self-reported directly by patients; it should be noted that a recall or knowledge bias may happen when answering clinical questions about their experience, especially those referring to treatments received or biomarkers; 5) a weakness of the DCE methodology which involves patients choosing between hypothetical treatment profiles with a set of attributes is that it may not reflect all the aspects of a treatment that can influence a patient’s preference; 6) although the sample size (*N* = 102) meets the minimum requirements for DCE analysis, we acknowledge that larger multinational cohorts would be desirable in a future to validate these findings in more diverse populations.

As such, results from this study may not reflect real-world treatment decisions, which could be influenced by other factors not captured in the survey, such as physician recommendation, and cost. These choices are designed to simulate real clinical decisions, but they do not carry the same clinical, financial, or emotional consequences as actual medical choices. Thus, differences can arise between stated and actual choices. Potential hypothetical bias was limited by constructing choice questions that mimic realistic clinical choices as closely as possible and map clearly into clinical evidence.

## Conclusions

Treatment efficacy was the top valued attribute across all patient segments and the second most important attribute was the risk of grade ≥ 3 adverse events (AE). Overall, therapies with better outcomes of PFS or AE grade 3 or higher would have a higher impact on the preference to choose a treatment from a patient perspective. The insights gained through this evidence-based study could help to support the ongoing development of patient-centered care in patients with advanced breast cancer. Our findings highlight the importance patients place on treatment efficacy and the risk of severe adverse events when making decisions. Incorporating patient preference data into clinical guidelines could help align recommended treatment pathways with what matters most to patients, thereby enhancing shared decision-making and adherence to therapy.

Future studies should aim to integrate discrete choice experiments (DCEs) in larger, more diverse patient populations and across different healthcare systems. Additionally, prospective clinical trials could incorporate patient preference endpoints to assess how these factors influence real-world treatment choices and outcomes. Such evidence would support the formal inclusion of patient-centered metrics into clinical decision frameworks and guideline updates.

## Supplementary Information

Supplementary Material 1.

Supplementary Material 2: Figure S1. A) Relative importance percentages add up to 100% across attributes for each respondent Bars represent the 95% CI for each attribute. B)Relative importance percentages add up to 100% across attributes for each respondent Bars represent the 95% CI for each attribute.

Supplementary Material 3: Supplemental Table 1. Competitive set used for relative treatment (1L) preference scores. Supplemental Table 2. Competitive set used for relative treatment (2L+) preference scores.

## Data Availability

Data are available upon request to data owner. Data access request should be sent to Dr. Xavier Guillaume, Associate Principal RWE, Oracle Life Sciences, xavier.guillaume@oracle.com.
