# Supplementary material for: Patient preferences for treatments in hormone receptor-positive/HER2-negative metastatic breast cancer in Italy: a discrete choice experiment study
Source: BMC Cancer. 2025 May 22;25:920. doi: 10.1186/s12885-025-14308-4 (PMC12101022; doi:10.1186/s12885-025-14308-4)
Supplement: Supplementary file 1 — Supplementary Material 1. [file 12885_2025_14308_MOESM1_ESM.docx]

| **Patient preferences of treatment attributes in Stage IV HR+/HER2- breast cancer** |
| --- |

| **Total Sample Size** | **Quotas** |
| --- | --- |
| 100 | ≥30 currently receiving their first or second line of hormonal therapy for the treatment of their metastatic breast cancer  **GROUP 1 FROM VARIABLE “TARGET”** |
|  | ≥30 expecting to receive or currently receiving their first chemotherapy for the treatment of their metastatic breast cancer  **GROUP 2 FROM VARIABLE “TARGET”** |
|  | ≥30 having completed at least one line of chemotherapy for the treatment of their metastatic breast cancer  **GROUP 3 FROM VARIABLE “TARGET”** |

**ONLINE DISCLAIMER:**

Cerner Enviza, an independent health research firm, is conducting an international survey to **better understand the perspective of people regarding health-related topics**. This survey is conducted on behalf of a pharmaceutical company (the ‘sponsor’) involved in Research and Development in breast cancer which name will be disclosed at the end of the survey. The survey outcome is meant to be published in scientific journals and communicated in scientific meetings.

We have a few questions to ask you to determine your qualification for this study. If qualified, the survey will take approximately 25 minutes to complete.

The qualification questions will just take 5 minutes. There are no right or wrong answers. We only want to learn some general information about you to determine if you qualify for the study. Please note that all responses will be kept strictly confidential.

**By participating in this survey, you understand and agree to the following:**

- I understand that this research is being conducted by Cerner Enviza in collaboration with Fieldcare on behalf of a pharmaceutical company (the ‘sponsor’). The name of the pharmaceutical company will be disclosed at the end of the survey.
- I understand that the aim of this research is to gain my views for research purposes AND IS NOT INTENDED AS A PROMOTIONAL EXERCISE.
- I understand that the outputs of this research will be used only for research purposes by the sponsor as part of ongoing disease education for healthcare professionals, the medical community, patient organizations, patients, and caregivers, and in research publications to advance understanding and increase disease awareness as well as treatment preferences of patients.
- I agree that anything I see or read during this research should be treated as confidential and should not be shared with any third party not involved in this study. Taking screenshots, sharing the material, or any action that would inform others of the study content is strictly prohibited.
- I understand that any information I disclose will be treated in the strictest confidence and the results of the research will be aggregated to provide an overall picture of attitudes related to the topics being covered in this survey. **No answers will be attributable to me as an individual**. My identity will remain confidential and none of my details will be passed on to any third party. My data will be stored on servers located in the EU.
- I understand that this survey will comply with ethical principles that are consistent with the Declaration of Helsinki and Good Pharmacoepidemiology Practices as well as General Data Protection Regulation (GDPR) and all applicable international/local data protection legislations.
- Taking part in this study is entirely my choice. The decision to participate in this research is entirely up to me. I have the right to refuse to answer any of the questions or end my participation at any time. If I withdraw my consent to participate in this survey, I will stop participating in this survey. Cerner Enviza S.A.S may continue to use the information obtained prior to my withdrawal request in order to maintain the legitimate interests of the study.
- I understand that the data for this survey will be stored, passed on, and analysed by Cerner Enviza S.A.S in a strictly anonymized form (my name or initials or other information linking my responses to me personally will be collected).
- I understand that no information will be collected that could directly identify me. I will not be mentioned by name in published reports about this survey or in other scientific publications or presentations.
- To comply with the sponsor’s regulatory obligations, the survey responses I will provide will be strictly anonymized, pooled with information from other participants, will only be shared with the survey Sponsor and will not be disclosed to any other party under any circumstance. My data will be stored for 5 years after the termination of this survey.
- If I have any questions, I will contact the recruitment/panel agency for this study.
- For more information about your rights and data retention period with Cerner Enviza, please see our privacy policy at <https://www.cernerenviza.com/privacy-notice> and our terms and conditions at <https://www.cernerenviza.com/terms-and-conditions>.
- The data collected in this survey will be used for publications in medical journals or congresses.

S0a. I confirm that I have read, understood and accept the points above, and am happy to proceed with the research survey on this basis.

Click on the following links for full Privacy Policy and Terms and Conditions

**[LINKS:
PRIVACY POLICY:** <https://www.cernerenviza.com/privacy-notice>
**TERMS & CONDITIONS:** <https://www.cernerenviza.com/terms-and-conditions> **]**

| **SELECT ONE** | | |
| --- | --- | --- |
| 1 | Accept |  |
| 2 | Decline | **TERMINATE & CLOSE** |

| **SCREENER** |
| --- |

**ASK EVERYONE**

S1. Please indicate your biological gender:

| 1 | Male |
| --- | --- |
| 2 | Female |
| 3 | I don’t know |

**NEW WEB PAGE**

**ASK EVERYONE**
S2. How old are you?

_____ years old  **[1-99]**

**TERMINATE IF <18**

**CREATE HIDDEN VARIABLE:**

**YRBIRTH=2022-S2**

**NEW WEB PAGE**

**ASK EVERYONE**

S3. Have you **ever** been diagnosed with the following types of cancer by a physician?

**[MULTIPLE ANSWERS - RANDOMIZE ITEMS]**

| 1 | Ovarian cancer |  |
| --- | --- | --- |
| 2 | Breast cancer | **MUST BE SELECTED TO CONTINUE** |
| 3 | Colorectal cancer |  |
| 4 | Gastric cancer |  |
| 5 | Lung cancer |  |
| 6 | None of the above **[EXCLUSIVE; ANCHOR]** | **TERMINATE** |

**TERMINATE IF CODES 1, 2, 3, 4 AND 5 ARE ALL SELECTED**

**NEW WEB PAGE**

**ASK EVERYONE**

S4. In what year were you diagnosed with breast cancer **for the first time**?

Year of diagnosis: _____  **[RANGE: YRBIRTH - 2022]**

**CREATE HIDDEN VARIABLE:**

**YRDIAGN=2022-S4**

**NEW WEB PAGE**

**ASK EVERYONE**

S5. Which of the following best describe the **current** stage of your breast cancer?

**SINGLE ANSWER**

| 1 | Stage 1:  The breast tumor is small and has not spread to lymph nodes | **TERMINATE** |
| --- | --- | --- |
| 2 | Stages 2 through 3a:  The breast tumor extended into the lymph nodes but not to the chest wall **[HOVER DEFINITION:** Your chest wall is made up of skin, fat, muscles, other tissues, and the rib cage and protects important organs such as your heart, lungs, and liver.**]** | **TERMINATE** |
| 3 | Stage 3b or 3c: The breast tumor has extended to the chest wall* but has not spread to other parts of the body distant from the breast | **TERMINATE** |
| 4 | Stage 4:  There were signs of breast cancer cells in parts of the body distant from the breast (metastases) | **CONTINUE** |
| 5 | I don't know | **TERMINATE** |

**NEW WEB PAGE**

**ASK EVERYONE**

S6. In what year were you diagnosed with **metastatic** breast cancer (which means that breast cancer cells have been identified in parts of your body distant from the breast)?

Year of diagnosis: _____  **[RANGE: YRDIAGN - 2022]**

**NEW WEB PAGE**

**ASK EVERYONE**

S7. Did your physician tell you that your breast cancer expresses extra amounts of **HER2 receptors**, meaning it is **HER2-positive (HER2+)**?

| 1 | Yes | **TERMINATE** |
| --- | --- | --- |
| 2 | No | **CONTINUE** |
| 3 | I don’t know | **CONTINUE** |

**NEW WEB PAGE**

**ASK EVERYONE**

S8. Have you **ever** received any of the following treatments for your breast cancer?

*Select all that apply*

**MULTIPLE ANSWERS**

| 1 | Phesgo® (pertuzumab/ trastuzumab/ hyaluronidase-zzxf) | **TERMINATE** |
| --- | --- | --- |
| 2 | Herceptin® (trastuzumab) |  |
| 3 | Herzuma® (trastuzumab-pkrb) |  |
| 4 | Kanjinti® (trastuzumab-anns) |  |
| 5 | Ogivri® (trastuzumab-dkst) |  |
| 6 | Ontruzant® (trastuzumab-dttb) |  |
| 7 | Trazimera® (trastuzumab-1yyp) |  |
| 8 | Trastuzumab |  |
| 9 | Enhertu® (trastuzumab deruxtecan) |  |
| 10 | Kadcyla® (T-DM1 or ado-trastuzumab emtansine) |  |
| 11 | Nerlynx® (neratinib) |  |
| 12 | Perjeta® (pertuzumab) |  |
| 13 | Tyverb® (lapatinib) |  |
| 14 | Tukysa® (tucatinib) |  |
| 15 | I don’t know | **CONTINUE** |
| 16 | None of the above | **CONTINUE** |

**NEW WEB PAGE**

**ASK EVERYONE**

S9. Did your physician tell you that your breast cancer is **estrogen and/or progesterone receptor positive** or **Hormone-receptor (HR) positive** (i.e., your cancer is fueled by hormones)?

**SINGLE ANSWER**

| 1 | Yes |
| --- | --- |
| 2 | No |
| 3 | I don’t know |

**NEW WEB PAGE**

**ASK EVERYONE**

S10. Have you **ever** received **hormonal therapy** (e.g., Anastrozole, Exemestane, Letrozole, Fulvestrant) for your breast cancer? Hormonal therapy is a type of treatment that adds, blocks, or removes hormones.

**SINGLE ANSWER**

| 1 | Yes |
| --- | --- |
| 2 | No |
| 3 | I don’t know |

**IF S9=1 OR S10=1 => CONTINUE**

**IF (S9=2 or 3) AND (S10=2 or 3) => TERMINATE**

**NEW WEB PAGE**

**ASK EVERYONE**

S11. Are you **currently** taking any treatment for your breast cancer?

**SINGLE ANSWER**

| 1 | Yes |
| --- | --- |
| 2 | No |

**ASK IF S11=1**

S11A. What is your **current** treatment for your breast cancer?

*Select all that apply*

**MULTIPLE ANSWERS**

| 1 | **Chemotherapy** **[HOVER DEFINITION:** Chemotherapy is a drug treatment that uses powerful chemicals to kill fast-growing cells in your body**]** (e.g., Capecitabine, Eribulin, Gemcitabine, Paclitaxel, Vinorelbine) |
| --- | --- |
| 2 | **Hormonal therapy** **[HOVER DEFINITION:** A type of treatment that adds, blocks, or removes hormones**]** (e.g., Anastrozole, Exemestane, Letrozole, Fulvestrant) |
| 3 | **Targeted therapy** **[HOVER DEFINITION:** A type of treatment that uses drugs to identify and attack specific types of cancer cells with less harm to normal cells**]** (e.g., Palbociclib, Abemaciclib, Ribociclib, Alpelisib, Everolimus, Olaparib, Talazoparib) |
| 4 | None of these **[EXCLUSIVE]** |
| 5 | Don’t know **[EXCLUSIVE]** |

**ASK IF S11=2**

S11B. What is the **next** treatment you are expecting to receive for your breast cancer?

*Select all that apply*

**MULTIPLE ANSWERS**

| 1 | **Chemotherapy** **[HOVER DEFINITION:** Chemotherapy is a drug treatment that uses powerful chemicals to kill fast-growing cells in your body**]** (e.g., Capecitabine, Eribulin, Gemcitabine, Paclitaxel, Vinorelbine) |
| --- | --- |
| 2 | **Hormonal therapy** **[HOVER DEFINITION:** A type of treatment that adds, blocks, or removes hormones**]** (e.g., Anastrozole, Exemestane, Letrozole, Fulvestrant) |
| 3 | **Targeted therapy** **[HOVER DEFINITION:** A type of treatment that uses drugs to identify and attack specific types of cancer cells with less harm to normal cells**]** (e.g., Palbociclib, Abemaciclib, Ribociclib, Alpelisib, Everolimus, Olaparib, Talazoparib) |
| 4 | None of these **[EXCLUSIVE]** |
| 5 | Don’t know **[EXCLUSIVE]** |

**NEW WEB PAGE**

**ASK EVERYONE**

S12. Since your cancer has been diagnosed metastatic, **how many different courses** of the following treatments did you receive until today **[SHOW IF S11=1:**, including your current treatment**]**? A course of treatment means the whole treatment plan made up of several cycles of treatment.

| 1 | **Chemotherapy** **[HOVER DEFINITION:** Chemotherapy is a drug treatment that uses powerful chemicals to kill fast-growing cells in your body**]** (e.g., Capecitabine, Eribulin, Gemcitabine, Paclitaxel, Vinorelbine) | **SINGLE ANSWER** | |
| --- | --- | --- | --- |
|  |  | 1 | 1 course of chemotherapy |
|  |  | 2 | 2 courses of chemotherapy |
|  |  | 3 | 3 courses or more of chemotherapy |
|  |  | 4 | **DO NOT SHOW IF CODE 1 IS SELECTED IN S11A:**  I never received chemotherapy since my cancer has been diagnosed metastatic |
| 2 | **Hormonal therapy** **[HOVER DEFINITION:** A type of treatment that adds, blocks, or removes hormones**]** (e.g., Anastrozole, Exemestane, Letrozole, Fulvestrant)  **IF S10=1** | **SINGLE ANSWER** | |
|  |  | 1 | 1 course of hormonal therapy |
|  |  | 2 | 2 courses of hormonal therapy |
|  |  | 3 | 3 courses or more of hormonal therapy |
|  |  | 4 | **DO NOT SHOW IF CODE 2 IS SELECTED IN S11A:**  I never received hormonal therapy since my cancer has been diagnosed metastatic |

**ASK IF S12.1<>4**

S13A. What chemotherapy drug(s) have you **ever** received since your cancer has been diagnosed **metastatic**? **[SHOW IF S11A=1:** Please include your current chemotherapy.**]**

*Select all that apply*

**MULTIPLE ANSWERS, RAMDOMIZE**

| 1 | Capecitabine |
| --- | --- |
| 2 | Eribulin |
| 3 | Gemcitabine |
| 4 | Paclitaxel |
| 5 | Vinorelbine |
| 6 | Other **[ANCHOR]** |
| 7 | Don’t know **[ANCHOR; EXCLUSIVE]** |

**ASK IF S12.2<>4**

S13B. What hormonal therapy drug(s) have you **ever** received since your cancer has been diagnosed **metastatic**? **[SHOW IF S11A=2:** Please include your current hormonal therapy.**]**

*Select all that apply*

**MULTIPLE ANSWERS, RAMDOMIZE**

| 1 | Anastrozole |
| --- | --- |
| 2 | Exemestane |
| 3 | Letrozole |
| 4 | Fulvestrant |
| 5 | Other **[ANCHOR]** |
| 6 | Don’t know **[ANCHOR; EXCLUSIVE]** |

**PROG: CREATE THE FOLLOWING HIDDEN VARIABLE “TARGET”:**

- **GROUP 1 (RECEIVING 1^ST^ OR 2^nd^ HT): S11A=2 AND S12.2=1 OR 2**
- **GROUP 2 (RECEIVING OR EXPECTING 1^ST^ CHEMO): (S11A=1 AND S12.1=1 (CURRENT 1^st^ CHEMO)) OR (S11A<>1 AND S11B=1 AND S12.1=4 (NEXT CHEMO WILL BE THE 1^st^ CHEMO))**
- **GROUP 3 (COMPLETED AT LEAST 1 CHEMO): (S12.1=2 OR 3) OR (S12.1=1 AND S11A<>1)**
- **OTHER => DO NOT FALL INTO ANY OF THE 3 GROUPS ABOVE**

**TERMINATE IF ‘OTHER’**

**CHECK QUOTAS**

**NEW WEB PAGE**

**IF TERMINATE, THEN SHOW TERMINATION TEXT:**

Thank you for your willingness to participate in this survey. Unfortunately, you do not meet the criteria we are looking for this research. Thank you again for your valuable time.

.

| **QUESTIONNAIRE** |
| --- |

**NEW WEB PAGE**

**SHOW EVERYONE WHO HAS PASSED THE SCREENER:**

Thank you for your interest in this survey! Your profile matches the survey eligibility criteria.

The purpose of this survey is to better understand perspectives of the potential benefits and risks associated with imaginary breast cancer therapies. This information will be helpful in understanding what is important to patients when considering treatment, and thereafter help physicians understand what treatment may be best for a patient, which might lead to better treatment results.

This survey contains 4 sections:

- Section A- Treatment characteristic ratings

This section asks you to rate treatment characteristics on a scale from 'Very Bad' to 'Very Good'.

- Section B- Trade-offs among treatment characteristics

This section involves a series of questions where you will be presented with two imaginary treatment profiles that differ on a few selected characteristics. You will be asked to read each profile carefully and then select the one you would most prefer.

- Section C- Clinical questions

This section contains questions about your health and the treatment for your breast cancer.

- Section D- Demographic questions

This section contains a few questions about your background, such as your employment status.

Please note that the survey can be completed using a computer or a tablet, not a smartphone. However, we recommend that you use a desktop or laptop computer for an optimal experience.

****** There are no right or wrong answers. We are interested in your opinion. If you cannot decide on an answer, please provide your best guess. ******

**NEW WEB PAGE**

**ASK EVERYONE**

Q0. In this survey, we will use percentages. Since not all people are familiar with percentages, we would like to explain what they are. Percentage means 'out of 100'. For example, 60% risk of diarrhea when taking a treatment means that out of 100 patients who received the treatment, 60 patients would experience diarrhea and 40 would not, as shown in the picture below:


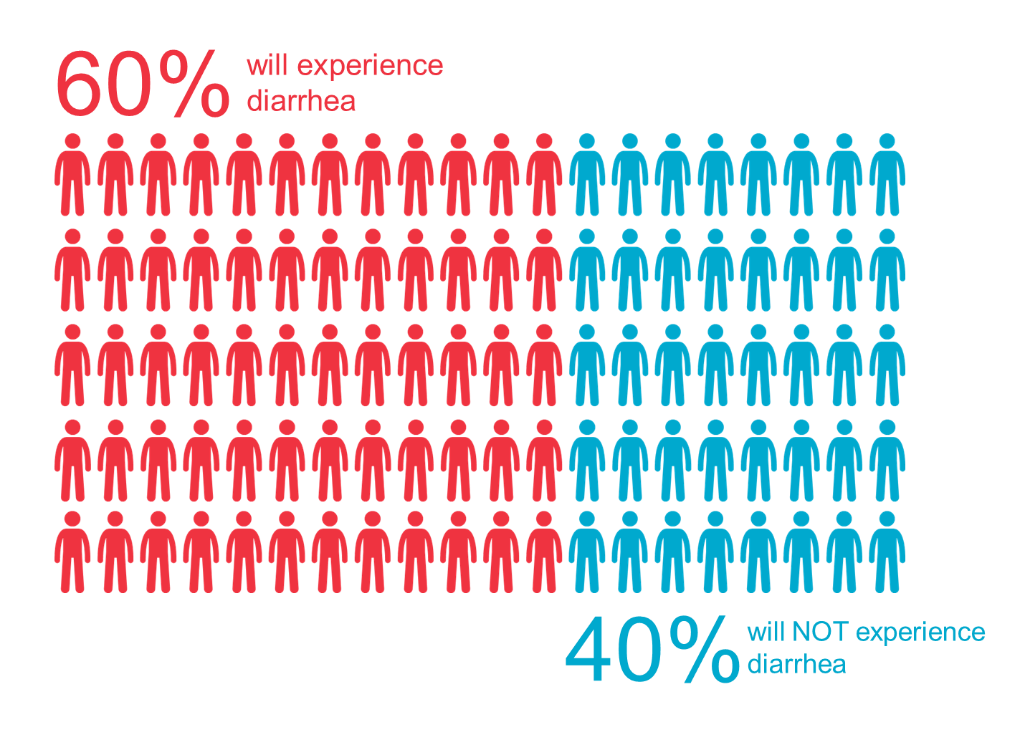


**NEW WEB PAGE**

**ASK EVERYONE**

Q1. Which of these represents 60%?

| 1 | 6 out of 100 |
| --- | --- |
| 2 | 60 out of 100 |
| 3 | 600 |
| 98 | I don’t know |

**IF CODE 2 SELECTED DISPLAY THE FOLLOWING TEXT**

You are correct, 60% means 60 out of 100.

**IF CODE 1, 3, 4 SELECTED DISPLAY THE FOLLOWING TEXT**

The correct answer was “60 out of 100”. A 60% risk of diarrhea when taking a treatment means 60 out of 100 patients taking the treatment will experience diarrhea and 40 patients will not.

**NEW WEB PAGE**

| **SECTION A: TREATMENT PLAN CHARACTERISTIC RATINGS** |
| --- |

To help you familiarize with the different treatment plan characteristics that will appear in this survey, the next few pages will show you these characteristics. Please rate each of them from ‘Very bad’ to ‘Very good’.

**Please note that the characteristics shown are referring to an imaginary treatment plan; they do not reflect any treatment that you may have taken in the past or are currently taking.**

**NEW WEB PAGE**

**ASK EVERYONE**

A1. The following characteristics are related to how well a treatment works (the average duration the cancer will not worsen). Please rate each of them from 'Very bad' to 'Very good'.

*Select ONE answer PER ROW*

| IMAGINARY TREATMENT CHARACTERISTICS | | **Very bad** | **Bad** | **Neither good nor bad (neutral)** | **Good** | **Very good** |
| --- | --- | --- | --- | --- | --- | --- |
| 1 | The cancer remains stable and does not worsen for an average of **3 months** | 1 | 2 | 3 | 4 | 5 |
| 2 | The cancer remains stable and does not worsen for an average of **11 months** | 1 | 2 | 3 | 4 | 5 |
| 3 | The cancer remains stable and does not worsen for an average of **28 months** | 1 | 2 | 3 | 4 | 5 |

**NEW WEB PAGE**

**ASK EVERYONE**

A2. Some cancer treatments may lead to a too much important **white blood cells reduction** which can cause infections. The following characteristics are related to the risk of low concentration in the blood of a type of white blood cells which normally help your body fight infections (also called neutropenia). Please rate each of them from 'Very bad' to 'Very good'.

*Select ONE answer PER ROW.*

| IMAGINARY TREATMENT CHARACTERISTICS | | **Very bad** | **Bad** | **Neither good nor bad (neutral)** | **Good** | **Very good** |
| --- | --- | --- | --- | --- | --- | --- |
| 1 | **2%** risk of **low concentration in the blood of a type of white blood cells** which normally help your body fight infections (any severity) | 1 | 2 | 3 | 4 | 5 |
| 2 | **44%** risk of **low concentration in the blood of a type of white blood cells** which normally help your body fight infections (any severity) | 1 | 2 | 3 | 4 | 5 |
| 3 | **88%** risk of **low concentration in the blood of a type of white blood cells** which normally help your body fight infections (any severity) | 1 | 2 | 3 | 4 | 5 |

**NEW WEB PAGE**

**ASK EVERYONE**

A3. The following characteristics related to the risk of hair loss (also called alopecia). Please rate each of from 'Very bad' to 'Very good'.

*Select ONE answer PER ROW.*

| IMAGINARY TREATMENT CHARACTERISTICS | | **Very bad** | **Bad** | **Neither good nor bad (neutral)** | **Good** | **Very good** |
| --- | --- | --- | --- | --- | --- | --- |
| 1 | **2%** risk of **hair loss** (any severity) | 1 | 2 | 3 | 4 | 5 |
| 2 | **20%** risk of **hair loss** (any severity) | 1 | 2 | 3 | 4 | 5 |
| 3 | **46%** risk of **hair loss** (any severity) | 1 | 2 | 3 | 4 | 5 |

**NEW WEB PAGE**

**ASK EVERYONE**

A4. The following characteristics related to the risk of vomiting. Please rate each of from 'Very bad' to 'Very good'.

*Select ONE answer PER ROW.*

| IMAGINARY TREATMENT CHARACTERISTICS | | **Very bad** | **Bad** | **Neither good nor bad (neutral)** | **Good** | **Very good** |
| --- | --- | --- | --- | --- | --- | --- |
| 1 | **10%** risk of **vomiting** (any severity) | 1 | 2 | 3 | 4 | 5 |
| 2 | **22%** risk of **vomiting** (any severity) | 1 | 2 | 3 | 4 | 5 |
| 3 | **41%** risk of **vomiting** (any severity) | 1 | 2 | 3 | 4 | 5 |

**NEW WEB PAGE**

**ASK EVERYONE**

A5. The following characteristics are related to the risk of diarrhea. Please rate each of them from 'Very bad' to 'Very good'.

*Select ONE answer PER ROW.*

| IMAGINARY TREATMENT CHARACTERISTICS | | **Very bad** | **Bad** | **Neither good nor bad (neutral)** | **Good** | **Very good** |
| --- | --- | --- | --- | --- | --- | --- |
| 1 | **12%** risk of **diarrhea** (any severity) | 1 | 2 | 3 | 4 | 5 |
| 2 | **58%** risk of **diarrhea** (any severity) | 1 | 2 | 3 | 4 | 5 |
| 3 | **90%** risk of **diarrhea** (any severity) | 1 | 2 | 3 | 4 | 5 |

**NEW WEB PAGE**

**ASK EVERYONE**

A6. The following characteristics related to the risk of a serious side effect. Please rate each of them from 'Very bad' to 'Very good'.

*Select ONE answer PER ROW.*

| IMAGINARY TREATMENT CHARACTERISTICS | | **Very bad** | **Bad** | **Neither good nor bad (neutral)** | **Good** | **Very good** |
| --- | --- | --- | --- | --- | --- | --- |
| 1 | **8%** risk of a **serious side effect** requiring medical intervention and possible hospitalization | 1 | 2 | 3 | 4 | 5 |
| 2 | **58%** risk of a **serious side effect** requiring medical intervention and possible hospitalization | 1 | 2 | 3 | 4 | 5 |
| 3 | **78%** risk of a **serious side effect** requiring medical intervention and possible hospitalization | 1 | 2 | 3 | 4 | 5 |

**NEW WEB PAGE**

**ASK EVERYONE**

A7. Please rate each of the following modes of treatment administration from 'Very bad' to 'Very good'.

*Select ONE answer PER ROW.*

| IMAGINARY TREATMENT CHARACTERISTICS | | **Very bad** | **Bad** | **Neither good nor bad (neutral)** | **Good** | **Very good** |
| --- | --- | --- | --- | --- | --- | --- |
| 1 | Daily oral pills taken indefinitely | 1 | 2 | 3 | 4 | 5 |
| 2 | Intravenous injection (into a vein) every 3 weeks taken indefinitely | 1 | 2 | 3 | 4 | 5 |
| 3 | Intravenous injection (into a vein) every 4 weeks taken indefinitely | 1 | 2 | 3 | 4 | 5 |
| 4 | Intramuscular injection (into muscle) every 4 weeks taken indefinitely | 1 | 2 | 3 | 4 | 5 |

**NEW WEB PAGE**

| **SECTION B: PREFERENCES (DCEs)** |
| --- |

IMPORTANT INFORMATION FOR NEXT QUESTION SERIES:

Different treatments may have different levels of effectiveness or risks of side effects. The purpose of this section is to understand your opinion about the importance of these differences. Based on your feedback, the medical community can better understand what aspects of treatments are important to people with metastatic breast cancer.

We will start with a set of questions where you will be asked to choose among 2 imaginary treatments that are taken as **a first treatment plan** after metastases (breast cancer cells in parts of your body distant from the breast) have been identified.

**Please imagine that you have not started treatment yet for metastatic cancer and are making the decision for the first time.**

Please note that this situation and the treatments presented are purely imaginary and do not intend to replicate your real situation nor the current treatments.

While reviewing the imaginary breast cancer treatment options, please assume:

- These treatment options are appropriate for you and are options you would be able to take.
- Any treatment outcomes not shown are the same between the treatment options.
- If you have received a breast cancer treatment, imagine that you have not started yet and are making the decision for the first time.

Please **review each treatment and choose the option you would prefer the most** in the circumstances described.

**BLOCK FOR 20 SECONDS**

**NEW WEB PAGE**

**ASK EVERYONE**

B_A0. Thinking about the time when you were diagnosed with metastatic breast cancer before starting any treatment, which treatment plan would you **most prefer**?

**[EXAMPLE TASK]**

| **Treatment A** | **Treatment B** |
| --- | --- |
| The cancer remains stable and does not worsen for an average of **13 months** | The cancer remains stable and does not worsen for an average of **28 months** |
| **2%** risk of **low concentration in the blood of a type of white blood cells** which normally help your body fight infections (any severity) | **44%** risk of **low concentration in the blood of a type of white blood cells** which normally help your body fight infections (any severity) |
| **33%** risk of **hair loss** (any severity) | **20%** risk of **hair loss** (any severity) |
| **30%** risk of **vomiting** (any severity) | **13%** risk of **vomiting** (any severity) |
| **50%** risk of **diarrhea** (any severity) | **82%** risk of **diarrhea** (any severity) |
| **58%** risk of a **serious side effect** requiring medical attention and possible hospitalization | **24%** risk of a **serious side effect** requiring medical attention and possible hospitalization |
| **Daily** oral pills taken indefinitely | **Daily** oral pills taken indefinitely |

| **SELECT ONE** | |
| --- | --- |
| 1 | Prefer Treatment A |
| 2 | Prefer Treatment B |

**PROG: BLOCK EACH TASK FOR 10 SECONDS**

**DCE-1 EXPERIMENTAL DESIGN WILL BE CREATED BASED ON ATTRIBUTES/LEVELS BELOW:**

| **Attribute** | **Attribute Description** | **Level 1** | **Level 2** | **Level 3** |
| --- | --- | --- | --- | --- |
| PFS | The cancer remains stable and does not worsen for an average of **XX months** | 13 months | 20 months | 28 months |
| Risk of Neutropenia | **XX%** risk of **low concentration in the blood of a type of white blood cells** which normally help your body fight infections (any severity) | 2% | 44% | 80% |
| Risk of Alopecia | **XX%** risk of **hair loss** (any severity) | 11% | 20% | 33% |
| Risk of Vomiting | **XX%** risk of **vomiting** (any severity) | 13% | 22% | 30% |
| Risk of Diarrhea | **XX%** risk of **diarrhea** (any severity) | 19% | 50% | 82% |
| Risk of Grade ¾ side effects | **XX%** risk of a **serious side effect** requiring medical attention and possible hospitalization | 24% | 58% | 78% |
| Mode of administration |  | **Daily** oral pills taken indefinitely | | |

**NEW WEB PAGE**

You are halfway done with this treatment choice task! Thank you so much for your attention!

We will now present you a different set of questions for other imaginary (hypothetical) treatments that are taken as later treatment options for metastatic breast cancer (i.e. after breast cancer cells have been identified in parts of the body distant from the breast).

The next set of questions will ask you to choose among 2 imaginary (hypothetical) treatments that are taken **after** the first treatment plan (i.e. second treatment plan, third treatment plan, etc.) for metastatic breast cancer.

Please keep in mind that this situation and the treatments presented are purely hypothetical and do not intend to replicate your real situation nor the current treatments.

**BLOCK FOR 15 SECONDS**

**NEW WEB PAGE**

**ASK EVERYONE**

B_B0. Thinking about a hypothetical situation where another treatment plan is prescribed to you after your first treatment plan for metastatic breast cancer to stop the cancer from progressing, which treatment plan would you **most prefer**?

**[EXAMPLE TASK]**

| **Treatment A** | **Treatment B** |
| --- | --- |
| The cancer remains stable and does not worsen for an average of **3 months** | The cancer remains stable and does not worsen for an average of **21 months** |
| **2%** risk of **low concentration in the blood of a type of white blood cells** which normally help your body fight infections (any severity) | **46%** risk of **low concentration in the blood of a type of white blood cells** which normally help your body fight infections (any severity) |
| **2%** risk of **hair loss** (any severity) | **27%** risk of **hair loss** (any severity) |
| **10%** risk of **vomiting** (any severity) | **41%** risk of **vomiting** (any severity) |
| **57%** risk of **diarrhea** (any severity) | **90%** risk of **diarrhea** (any severity) |
| **41%** risk of a **serious side effect** requiring medical attention and possible hospitalization | **76%** risk of a **serious side effect** requiring medical attention and possible hospitalization |
| **Intramuscular** injection (into muscle) every 4 weeks taken indefinitely | **Intravenous** injection (into a vein) every 3 weeks taken indefinitely |

| **SELECT ONE** | |
| --- | --- |
| 1 | Prefer Treatment A |
| 2 | Prefer Treatment B |

**PROG: BLOCK EACH TASK FOR 10 SECONDS**

**DCE-2 EXPERIMENTAL DESIGN WILL BE CREATED BASED ON ATTRIBUTES/LEVELS BELOW:**

| **Attribute** | **Attribute Description** | **Level 1** | **Level 2** | **Level 3** | **Level 4** |
| --- | --- | --- | --- | --- | --- |
| PFS | The cancer remains stable and does not worsen for an average of **XX months** | 3 months | 11 months | 21 months |  |
| Risk of Neutropenia | **XX%** risk of **low concentration in the blood of a type of white blood cells** which normally help your body fight infections (any severity) | 2% | 46% | 88% |  |
| Risk of Alopecia | **XX%** risk of **hair loss** (any severity) | 2% | 27% | 46% |  |
| Risk of Vomiting | **XX%** risk of **vomiting** (any severity) | 10% | 26% | 41% |  |
| Risk of Diarrhea | **XX%** risk of **diarrhea** (any severity) | 12% | 57% | 90% |  |
| Risk of Grade ¾ side effects | **XX%** risk of a **serious side effect** requiring medical attention and possible hospitalization | 8% | 41% | 76% |  |
| Mode of administration | - | **Daily** oral pills taken indefinitely | **Intravenous** injection (into a vein) every 3 weeks taken indefinitely | **Intravenous** injection (into a vein) every 4 weeks taken indefinitely | **Intramuscular** injection (into muscle) every 4 weeks taken indefinitely |

**NEW WEB PAGE**

| **SECTION C: CLINICAL QUESTIONS** |
| --- |

The following questions will be about your health and the treatment for your breast cancer, and then about your background, such as your employment status.

**NEW WEB PAGE**

**ASK EVERYONE**

C1. Which of the following side-effects related to your treatment for your breast cancer have you ever experienced?

*Select all that apply*

| **SELECT ALL THAT APPLY**  **RANDOMIZE ITEMS** | |  |
| --- | --- | --- |
| 1 | Low concentration in the blood of a type of white blood cells which normally help your body fight infections (neutropenia) | **ASK C1A** |
| 2 | Alopecia (hair loss) | **ASK C1B** |
| 3 | Vomiting | **ASK C1C** |
| 4 | Diarrhea | **ASK C1D** |
| 99 | None of these **[EXCLUSIVE; ANCHOR]** | **SKIP TO C2** |

**ASK C1A IF C1= 1**

C1A. How bad was the **worst** neutropenia (low concentration in the blood of a type of white blood cells which normally help your body fight infections) you have **ever** experienced because of your treatment for your breast cancer?

| **SELECT ONE** | | | | |
| --- | --- | --- | --- | --- |
| **Not at all bad** | **A little bad** | **Moderately bad** | **Very bad** | **Extremely bad** |
| 1 | 2 | 3 | 4 | 5 |

**ASK C1B IF C1= 2**

C1B. How bad was the **worst** hair loss you have **ever** experienced because of your treatment for your breast cancer?

| **SELECT ONE** | | | | |
| --- | --- | --- | --- | --- |
| **Not at all bad** | **A little bad** | **Moderately bad** | **Very bad** | **Extremely bad** |
| 1 | 2 | 3 | 4 | 5 |

**ASK C1C IF C1= 3**

C1C. How bad was the **worst** vomiting you have **ever** experienced because of your treatment for your breast cancer?

| **SELECT ONE** | | | | |
| --- | --- | --- | --- | --- |
| **Not at all bad** | **A little bad** | **Moderately bad** | **Very bad** | **Extremely bad** |
| 1 | 2 | 3 | 4 | 5 |

**ASK C1D IF C1= 4**

C1D. How bad was the **worst** diarrhea you have **ever** experienced because of your treatment for your breast cancer?

| **SELECT ONE** | | | | |
| --- | --- | --- | --- | --- |
| **Not at all bad** | **A little bad** | **Moderately bad** | **Very bad** | **Extremely bad** |
| 1 | 2 | 3 | 4 | 5 |

**NEW WEB PAGE**

**ASK EVERYONE**

C2. Have you ever been hospitalized because of side-effects related to your treatment for your breast cancer?

| **SELECT ONE** | |
| --- | --- |
| 1 | Yes |
| 2 | No |

**NEW WEB PAGE**

C3. Have you ever experienced a side-effect related to your treatment for your breast cancer leading to your treatment being stopped?

| **SELECT ONE** | |
| --- | --- |
| 1 | Yes |
| 2 | No |

**NEW WEB PAGE**

**ASK EVERYONE**

C4. Thinking of your **current health state**, with which of the following statement would you agree? Please choose the answer that describes best your usual situation.

| **SELECT ONE** | |
| --- | --- |
| 1 | I am fully active, able to carry on full activities without restriction |
| 2 | I am restricted in physically strenuous activity, can walk, able to carry out light housework |
| 3 | I am ambulatory and capable of all self-care but unable to carry out any work activities, up and about >50% of waking hours |
| 4 | I need some help taking care of self; spend more than half day in bed or chair |
| 5 | I cannot take care of self at all; spend all day in bed or chair |

**NEW WEB PAGE**

**ASK IF S1 = 2**

C5. How would you describe your **current menstrual status**?

| **SELECT ONE** | |
| --- | --- |
| 1 | I am still having my usual periods |
| 2 | I experienced changes in my periods but have not gone 12 months in a row without a period |
| 3 | I am menopausal (I have gone longer than 12 months ago without a period) due to my cancer treatment |
| 4 | I am menopausal because of age |
| 98 | Don’t know |

**NEW WEB PAGE**

| **SECTION D: DEMOGRAPHICS** |
| --- |

**ASK EVERYONE**

D1. What is your **current** marital status?

| **SELECT ONE** | |
| --- | --- |
| 1 | Married / Living with partner |
| 2 | Single |
| 99 | Decline to answer |

**NEW WEB PAGE**

**ASK EVERYONE**

D2. How **many children** under the age of 18 live in your household?

_______ children **[RANGE: 0 - 15]**

**NEW WEB PAGE**

**ASK D3 IF D2>0**

D3. For how many of those children under the age of 18 that live in your household are you a parent or a legal guardian?

_______ children **[RANGE: 0 - ANSWER of D2]**

**NEW WEB PAGE**

**ASK EVERYONE**

D4. What is the highest level of formal **education** you have completed?

| **SELECT ONE** | |
| --- | --- |
| 1 | Less than high school |
| 2 | High school |
| 3 | College/university |
| 4 | Postgraduate |
| 99 | Decline to answer |

**NEW WEB PAGE**

**ASK EVERYONE**

D5. What is your **current** employment status?

| **SELECT ONE** | |
| --- | --- |
| 1 | Employed (full time) |
| 2 | Employed (part time) |
| 3 | Employed, but currently on temporary leave of absence or long-term disability |
| 4 | Not employed |
| 5 | Retired |
| 98 | Other |

**NEW WEB PAGE**

**ASK EVERYONE**

D6. **Before** you were diagnosed with breast cancer, what was your employment status?

| **SELECT ONE** | |
| --- | --- |
| 1 | Employed (full time) |
| 2 | Employed (part time) |
| 3 | Employed, but currently on temporary leave of absence or long-term disability |
| 4 | Not employed |
| 5 | Retired |
| 98 | Other |

**NEW WEB PAGE**

**ASK EVERYONE**

D7. Which of the following best describes the location where you live?

| **SELECT ONE** | |
| --- | --- |
| 1 | Urban area |
| 2 | Suburban area |
| 3 | Rural area |

**NEW WEB PAGE**

**Thank you for taking the time to complete this survey! Your answers have now been submitted. The sponsors of this study are Daiichi Sankyo and AstraZeneca.**
