# Supplementary material for: Patient preferences for treatments in hormone receptor-positive/HER2-negative metastatic breast cancer in Italy: a discrete choice experiment study
Source: BMC Cancer. 2025 May 22;25:920. doi: 10.1186/s12885-025-14308-4 (PMC12101022; doi:10.1186/s12885-025-14308-4)
Supplement: Supplementary file 2 — Supplementary Material 2: Figure S1. A) Relative importance percentages add up to 100% across attributes for each respondent Bars represent the 95% CI for each attribute. B)Relative importance percentages add up to 100% across attributes for each respondent Bars represent the 95% CI for each attribute. [file 12885_2025_14308_MOESM2_ESM.pdf]

Figure S1

A

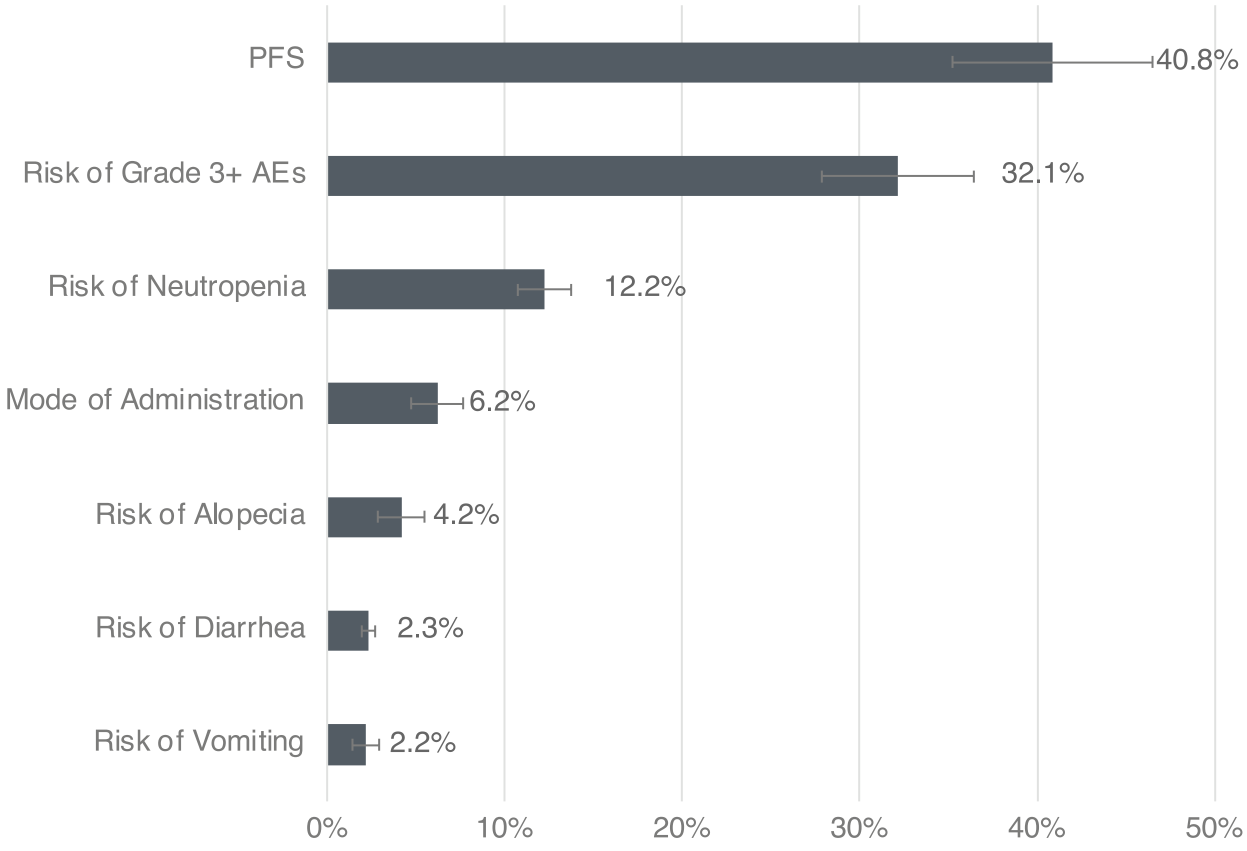

Relative importance percentages add up to 100% across attributes for each respondent
Bars represent the 95% CI for each attribute

B

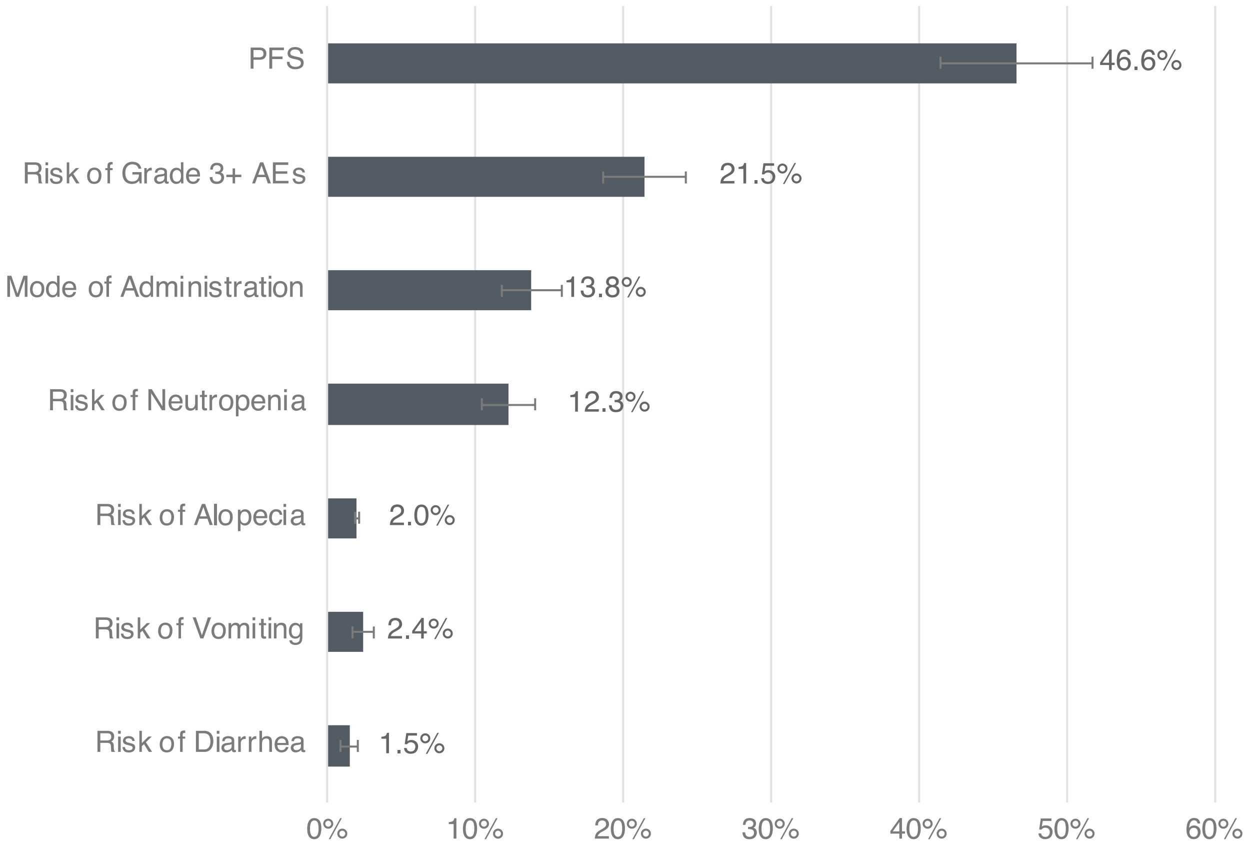

Relative importance percentages add up to 100% across attributes for each respondent
Bars represent the 95% CI for each attribute
